# Time-resolved photoluminescence studies of annealed 1.3-μm GaInNAsSb quantum wells

**DOI:** 10.1186/1556-276X-9-81

**Published:** 2014-02-17

**Authors:** Michal Baranowski, Robert Kudrawiec, Marcin Syperek, Jan Misiewicz, Tomas Sarmiento, James S Harris

**Affiliations:** 1 Institute of Physics, Wroclaw University of Technology, Wybrzeze Wyspianskiego 27, Wroclaw 50-370, Poland; 2 Solid State and Photonics Laboratory, Stanford University, Stanford, CA 94305-4075, USA

**Keywords:** GaInNAsSb, Quantum wells, Time-resolved spectroscopy

## Abstract

Time-resolved photoluminescence (PL) was applied to study the dynamics of carrier recombination in GaInNAsSb quantum wells (QWs) emitting near 1.3 μm and annealed at various temperatures. It was observed that the annealing temperature has a strong influence on the PL decay time, and hence, it influences the optical quality of GaInNAsSb QWs. At low temperatures, the PL decay time exhibits energy dependence (i.e., the decay times change for different energies of emitted photons), which can be explained by the presence of localized states. This energy dependence of PL decay times was fitted by a phenomenological formula, and the average value of *E*_0_, which describes the energy distribution of localized states, was extracted from this fit and found to be smallest (*E*_0_ = 6 meV) for the QW annealed at 700°C. In addition, the value of PL decay time at the peak energy was compared for all samples. The longest PL decay time (600 ps) was observed for the sample annealed at 700°C. It means that based on the PL dynamics, the optimal annealing temperature for this QW is approximately 700°C.

## Background

Incorporation of small amounts of nitrogen into a GaInAs host causes a strong reduction of the energy gap [1] as well as a reduction of the lattice constant. A few percent of nitrogen is enough to tune the energy gap of GaInNAs to the 1.3- and 1.55-μm spectral regions. Because of that, GaInNAs alloys have attracted much attention for low-cost GaAs-based lasers operating at II and III telecommunication windows [2-4]. However, the optical quality of Ga(In)NAs alloys strongly deteriorates with increasing nitrogen concentration due to phase segregation and the incorporation of point defects such as gallium interstitials [5], nitrogen interstitials [6,7], arsenic antisites [6], and gallium vacancies [6]. Post-growth annealing is the standard procedure to remove defects in an as-grown material to improve its optical quality [8,9]. The optical quality of strained GaInNAs alloys can also be improved by adding antimony to form GaInNAsSb alloys with 2% to 3% Sb concentration. This is due to the reactive surfactant properties of antimony, which reduce the group III surface diffusion length suppressing phase segregation and roughening and thereby improving alloy homogeneity [10,11]. The incorporation of antimony reduces the energy gap of the alloy, and hence, it is possible to reach longer emission wavelengths with lower nitrogen concentrations. Using GaInNAsSb quantum wells (QWs), lasers and vertical-cavity surface-emitting lasers operating at 1.3 μm [12] and 1.55 μm [13,14] have been demonstrated. However, the quality of an as-grown GaInNAsSb material can still be improved by post-growth annealing [15,16]. The effects of annealing on the optical properties of GaInNAsSb QWs have been studied in detail (see, for example, [13] and references therein). The annealing conditions for dilute nitrides are optimized based on the peak or integrated photoluminescence (PL) intensity. Recently, we demonstrated that the peak PL intensity in 1.3-μm GaInNAsSb QWs depends not only on the optical quality of the QW but also on the efficiency of carrier collection of the QW [17]. In this paper, we applied time-resolved photoluminescence (TRPL) to investigate the carrier dynamics in GaInNAsSb QWs at low temperature and identify the optimal annealing conditions based on the parameters that describe the carrier dynamics.

## Methods

The QW structures used in this study were grown by molecular beam epitaxy on (001) n-type GaAs substrates and consist of a 300-nm GaAs buffer layer, a 7.5-nm Ga_0.66_In_0.34_ N_0.008_As_0.97_Sb_0.022_ QW surrounded by 20-nm strain-compensating GaN_0.008_As_0.992_ barriers, and a 50-nm GaAs cap layer. It is worth noting that GaN_0.008_As_0.992_ barriers do not compensate the strain in the QW region, but they help improve the structural quality of the Ga_0.66_In_0.34_ N_0.008_As_0.97_Sb_0.022_ layer. After the growth, the samples were annealed for 60 s at different temperatures from 680°C to 800°C in 20°C steps. The growth conditions are similar to those used for a 1.55-μm GaInNAsSb QW and can be found elsewhere [18]. For the TRPL experiment, the samples were held in a vapor helium cryostat allowing measurements at variable temperatures. They were excited by a mode-locked Ti:sapphire laser with a 76-MHz repetition rate and a pulse duration of 150 fs. The laser wavelength was set to 800 nm and its average excitation power density was approximately 3 W/cm^2^. The PL signal was dispersed by a 0.3-m-focal length monochromator, and the temporal evolution of the PL signal was detected by a streak camera with S1 photocathode while the time-integrated spectrum was recorded by an InGaAs CCD camera. The effective time resolution of the system is approximately 20 ps.

## Results and discussion

**Figure 1:**
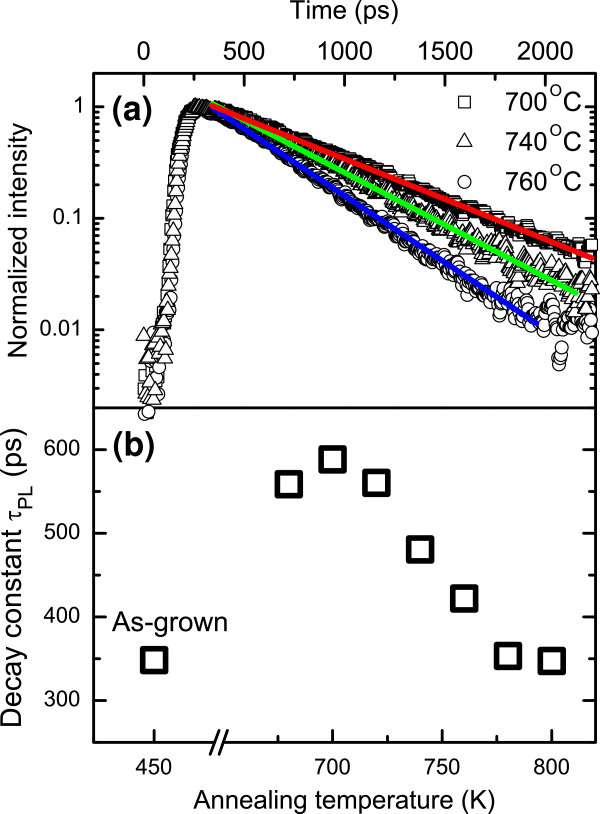
**PL decay curves and decay time constants. (a)** PL decay curves (taken at the maximum of PL emission) for samples annealed at three different temperatures. There is a clearly visible influence of the annealing temperature on the decay rate. Lines represent single exponential fit. **(b)** Decay time constants for all structures.

Figure 1a shows the temporal evolution of the PL signal from the samples annealed at various temperatures taken at the peak energy of the PL spectrum at *T* = 5 K. The decay curves can be very well fitted by a single exponential decay: *I* ~ exp(*t* / *τ*_PL_), where *τ*_PL_ is the PL decay time constant.

Figure 1b shows *τ*_PL_ constants extracted by fitting the experimental data. It is clearly visible that the annealing temperature has a significant influence on the PL decay time. The *τ*_PL_ equals approximately 350 ps for the as-grown QW and increases after annealing to 600 ps for the QW annealed at 700°C. At higher annealing temperatures, *τ*_PL_ decreases with increasing annealing temperature reaching values comparable to the *τ*_PL_ of the as-grown QW for annealing temperatures in the 780°C to 800°C range.

The *τ*_PL_ constant is directly related to the optical quality of QW since *τ*_PL_ can be expressed in terms of the radiative (*τ*_r_) and nonradiative (*τ*_nr_) lifetimes according to the formula 1 / *τ*_PL_ = 1 / *τ*_r_ + 1 / *τ*_nr_. The radiative lifetime is proportional to the wave function overlap which does not change significantly during annealing. Obviously, the annealing can cause some QW intermixing [19,20], but this change in QW potential shape is too small to significantly reduce the wave function overlap. Therefore, any differences in *τ*_PL_ arise from differences in *τ*_nr_. Stronger nonradiative recombination leads to shorter *τ*_nr_ and hence shorter *τ*_PL_. From the TRPL studies (see Figure 1), we can conclude that the optimal annealing temperature (in the sense of the optical quality of the QW layer) is approximately 700°C as it yields the longest *τ*_PL_. Annealing at higher temperatures creates defects that act as new centers of nonradiative recombination that degrade the optical quality of the QW. This conclusion is consistent with our room-temperature TRPL studies for this set of samples [17]. It is worth noting that the low-temperature TRPL measurements presented in this work were performed at a relatively low excitation power density (3 W/cm^2^) to minimize the saturation of the localized states [21], which can obscure the differences between the samples annealed at different temperatures.

**Figure 2:**
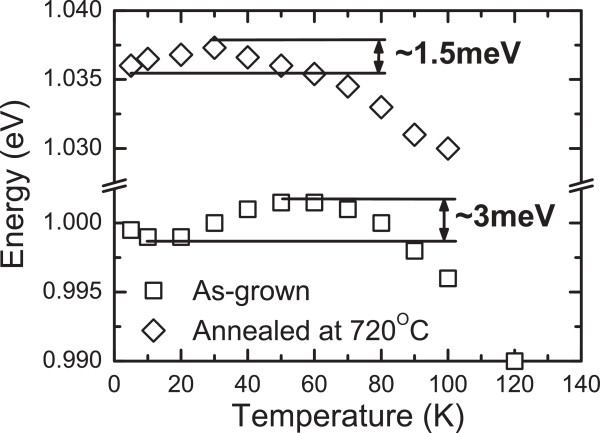
Dependence of PL peak maximum vs. temperature for as-grown (square) and annealed (720°C) (diamond) GaInNAsSb QW samples.

**Figure 3:**
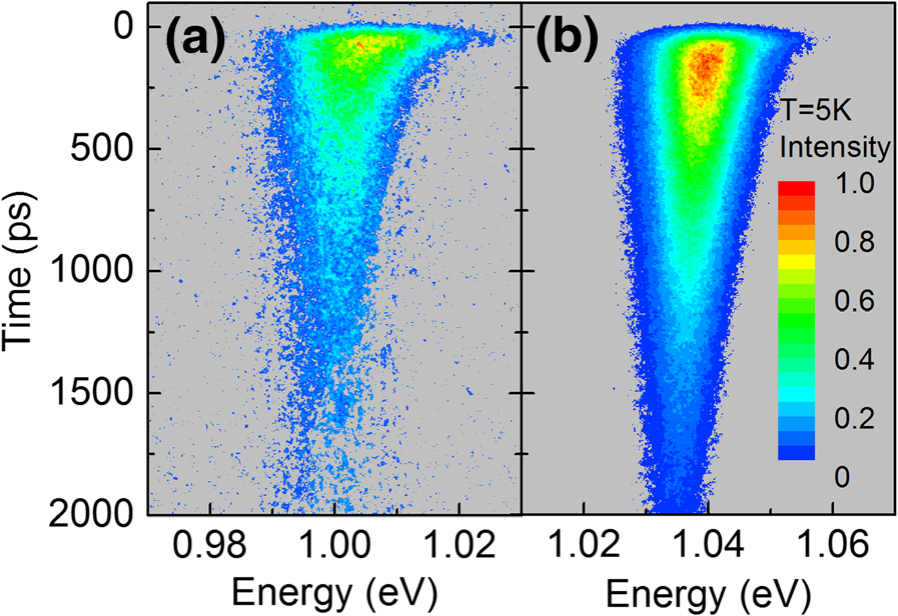
Temporal evolution of PL spectrum (i.e., streak image) for (a) as-grown and (b) annealed (720°C) GaInNAsSb QW samples.

**Figure 4:**
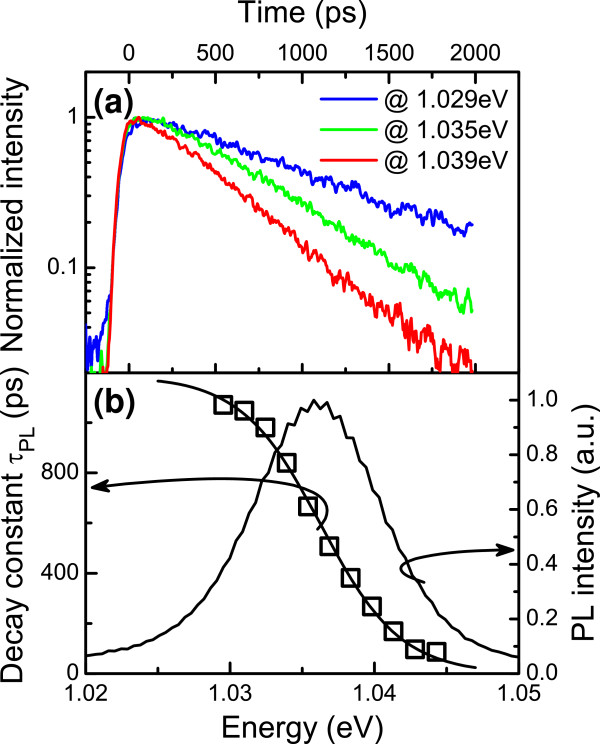
**Temporal evolution of PL intensity and dependence of decay time constant. (a)** Temporal evolution of PL intensity at different energies of detection. **(b)** Dependence of decay time constant versus energy together with time-integrated TRPL spectra.

**Figure 5:**
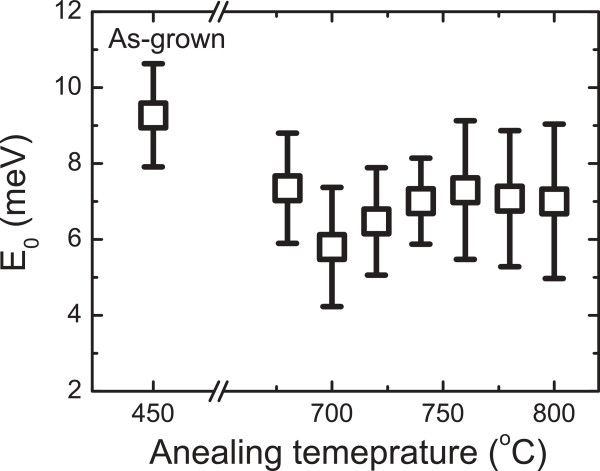
**Average energy of localized states *E***_**0 **_**as a function of annealing temperature.**

Despite the fact that antimony improves the homogeneity of GaInNAsSb QWs, we found evidence of carrier localization in the investigated QW structures at low temperatures. Figure 2 shows the temperature dependence of the peak PL energy for the as-grown and annealed GaInNAsSb QWs (obtained under pulse excitation with an average excitation power density of 3 W/cm^2^). The observed higher emission energies for the annealed QW are due to a rearrangement of the nitrogen nearest-neighbor environment upon annealing [22,23]. In both cases, we observe an S shape (but it is much stronger for the as-grown sample) in the temperature dependence of the peak PL energy, which is characteristic of a system where carrier localization is present [24-27]. The initial redshift is caused by a redistribution of excitons over deep localized states, while the blueshift is due to the escape of excitons to delocalized states (blueshift). The further redshift of the peak PL energy follows the reduction of energy gap with temperature. Changes in peak PL energy are stronger for the as-grown sample than for the annealed sample (see Figure 2). As we can see, annealing reduces the blueshift of the PL peak at low temperature, which means that annealing reduces the density of localized states and/or reduces their localization energy. The presence of localized states also has a significant impact on the dynamics of PL at low temperature causing the PL decay times to be longer on the low-energy side than on the high-energy side. Figure 3 shows the temporal evolution of the PL spectrum (i.e., streak image) for (a) as-grown and (b) annealed (720°C) GaInNAsSb QWs. The characteristic feature of PL dynamics in dilute nitride [24,28] and other [29-33] QW systems with localization effects (i.e., strong asymmetry of PL decay time at 5 K) is visible in both cases, but it is stronger for the as-grown sample. An example of the detailed analysis of PL decays at different energies is presented in Figure 4a,b. We can see that the PL decay at the high-energy side is faster than that at the low-energy side changing from approximately 100 ps to approximately 1,000 ps. This effect is due to the carrier localization as is the S-shaped temperature dependence of the PL peak energy. Exciton trapping and transfer between different localized states cause the PL decay time to change with the emission energy [26,34]. The values of *τ*_PL_ are reduced at higher energies because the exciton recombination dynamics are affected by the energy transfer process to lower energy states. Simultaneously, the exciton transfer from low energy states to high energy states is damped since excitons do not have sufficient thermal energy for such a transfer. Due to this asymmetry of exciton hopping rate between low and high energy localizing states, the *τ*_PL_ at the low-energy side is elongated due to refilling of states by relaxing excitons. The theoretical simulation of PL spectra presented in the literature indicates that the density of states is proportional to exp(-*E/E*_0_) in dilute nitride structures [35-38]. In such case, the energy dependence of the PL decay time can be described by the following formula [34]:

(1) $$\tau_{\text{PL}}\left(E\right)=\frac{\tau_{\text{rad}}}{1+\left\{\exp\left(E-E_{m}\right)/E_{0}\right\}}$$

where *E*_0_ is an average energy for the density of states, *τ*_rad_ is the maximum radiative lifetime, and *E*_m_ is defined as the energy where the recombination rate equals the transfer rate [26,34,39]. The obtained energy dependence of the PL decay time can by very well fitted by Equation 1 as shown in Figure 4b. Using this approach to analyze TRPL data, we are able to extract the *E*_0_ parameter which describes the distribution of localized states. The fits of experimental data to Equation 1 are shown in Figure 5. It is observed that the value of the *E*_0_ parameter is clearly higher for the as-grown QW than for the annealed QWs. Increasing the annealing temperature up to 700°C reduces the average energy of localized states *E*_0_ up to 6 meV. As the annealing temperature is further increased, *E*_0_ starts to increase due to degradation of the optical quality of the QW. This means that annealing not only reduces the density of localized states but also changes the average energy distribution of these states. Despite the large uncertainty in the values of the *E*_0_ parameter, its dependence on annealing temperature correlates well with the dependence on annealing temperature of the PL decay time at the peak PL energy (see Figure 1). The smallest value of the average localization energy *E*_0_ is observed for the sample annealed at 700°C which is characterized by the longest decay time. This means that annealing reduces both the number of nonradiative recombination centers and the deepness of localizing states.

The values of *E*_0_ for the annealed 1.3-μm GaInNAsSb QWs are in the range of 6 to 7 meV. These values are comparable to the values of *E*_0_ for dilute nitrides reported in the literature: approximately 6 meV for a GaInNAs multiple QW structure with 1.5% of nitrogen [26] and approximately 9 meV for a GaInNAs epilayer with 1% of nitrogen [28].

## Conclusions

In conclusion, 1.3-μm GaInNAsSb QWs annealed at various temperatures (from 680°C to 800°C in 20°C steps) were studied by low-temperature TRPL. It has been shown that exciton dynamics in these QWs change significantly with annealing temperature. Due to carrier localization, strong energy dependence of the PL decay time is observed for all samples at low temperatures. This energy dependence was fitted by a phenomenological formula that assumes an exponential distribution of localized states. The average value of *E*_0_, which describes the energy distribution of localized states, has been extracted from this fit, and its dependence on annealing temperature was studied. The smallest value of *E*_0_ was observed for the GaInNAsSb QW annealed at 700°C. In addition, the PL decay time measured at the peak PL energy was compared for all samples. The longest PL decay time was also observed for the QW annealed at 700°C. Based on these parameters that describe the carrier dynamics at low temperature, it can be concluded that the optimal annealing temperature for this QW is approximately 700°C.

## Abbreviations

PL: photoluminescence; QWs: quantum wells; TRPL: time-resolved photoluminescence.

## Competing interests

The authors declare that they have no competing interests.

## Authors’ contributions

MB wrote this article and made substantial contributions to the acquisition of data. RK contributed to the analysis and interpretation of data. MS contributed to the acquisition of data. JM has been involved in drafting the manuscript. TS and JSH performed the MBE growth and annealing of the investigated QW structures and contributed to the manuscript preparation. All authors read and approved the final manuscript.

## References

[B1] Shan W Walukiewicz W Ager JW Haller EE Geisz JF Friedman DJ Olson JM Kurtz SR Band anticrossing in GaInNAs alloys Phys Rev Lett 1999 9 1221 1224 10.1103/PhysRevLett.82.1221

[B2] Choquette KD Klem JF Fischer AJ Blum O Allerman AA Fritz IJ Kurtz SR Breiland WG Sieg R Geib KM Scott JW Naone RL Room temperature continuous wave InGaAsN quantum well vertical-cavity lasers emitting at 1.3 μm Electron Lett 2000 9 1388 10.1049/el:20000928

[B3] Tansu N Mawst LJ Temperature sensitivity of 1300-nm InGaAsN quantum-well lasers IEEE Photonics Technol Lett 2002 9 1052 1054

[B4] Jaschke G Averbeck R Geelhaar L Riechert H Low threshold InGaAsN/GaAs lasers beyond 1500 nm J Cryst Growth 2005 9 224 228 10.1016/j.jcrysgro.2004.12.059

[B5] Wang XJ Puttisong Y Tu CW Ptak AJ Kalevich VK Egorov AY Geelhaar L Riechert H Chen WM Buyanova IA Dominant recombination centers in Ga(In)NAs alloys: Ga interstitials Appl Phys Lett 2009 9 241904 10.1063/1.3275703

[B6] Chen WM Buyanova IA Tu CW Defects in dilute nitrides: significance and experimental signatures Optoelectron IEE Proc 2004 9 379 384 10.1049/ip-opt:20040939

[B7] Krispin P Gambin V Harris JS Ploog KH Nitrogen-related electron traps in Ga(As, N) layers (≤3% N) J Appl Phys 2003 9 6095 6099 10.1063/1.1568523

[B8] Spruytte SG Coldren CW Harris JS Wampler W Krispin P Ploog K Larson MC Incorporation of nitrogen in nitride-arsenides: origin of improved luminescence efficiency after anneal J Appl Phys 2001 9 4401 4406 10.1063/1.1352675

[B9] Pan Z Li LH Zhang W Lin YW Wu RH Ge W Effect of rapid thermal annealing on GaInNAs/GaAs quantum wells grown by plasma-assisted molecular-beam epitaxy Appl Phys Lett 2000 9 1280 1282 10.1063/1.1289916

[B10] Yang X Jurkovic MJ Heroux JB Wang WI Molecular beam epitaxial growth of InGaAsN:Sb/GaAs quantum wells for long-wavelength semiconductor lasers Appl Phys Lett 1999 9 178 180 10.1063/1.124311

[B11] Massies J Grandjean N Surfactant effect on the surface diffusion length in epitaxial growth Phys Rev B 1993 9 8502 8505 10.1103/PhysRevB.48.8502 10007060

[B12] Shimizu H Setiagung C Ariga M Ikenaga Y Kumada K Hama T Ueda N Iwai N Kasukawa A 1.3-μm-range GaInNAsSb-GaAs VCSELs IEEE J Sel Top Quantum Electron 2003 9 1214 1219 10.1109/JSTQE.2003.819505

[B13] Bank SR Bae H Goddard LL Yuen HB Wistey MA Kudrawiec R Harris JS Recent progress on 1.55-μm dilute-nitride lasers IEEE J Quantum Electron 2007 9 773 785

[B14] Sarmiento T Bae HP O'Sullivan TD Harris JS GaAs-based 1.53 μm GaInNAsSb vertical cavity surface emitting lasers Electron Lett 2009 9 978 10.1049/el.2009.1626

[B15] Kudrawiec R Poloczek P Misiewicz J Bae HP Sarmiento T Bank SR Yuen HB Wistey MA Harris JS Jr Contactless electroreflectance of GaInNAsSb/GaNAs/GaAs quantum wells emitting at 1.5–1.65 μm: broadening of the fundamental transition Appl Phys Lett 2009 9 031903 10.1063/1.3073718

[B16] Bae HP Bank SR Yuen HB Sarmiento T Pickett ER Wistey MA Harris JS Temperature dependencies of annealing behaviors of GaInNAsSb/GaNAs quantum wells for long wavelength dilute-nitride lasers Appl Phys Lett 2007 9 231119 10.1063/1.2746944

[B17] Baranowski M Kudrawiec R Latkowska M Syperek M Misiewicz J Sarmiento T Harris JS Enhancement of photoluminescence from GaInNAsSb quantum wells upon annealing: improvement of material quality and carrier collection by the quantum well J Phys Condens Matter 2013 9 065801 10.1088/0953-8984/25/6/065801 23306016

[B18] Harris JS Jr Kudrawiec R Yuen HB Bank SR Bae HP Wistey MA Jackrel D Pickett ER Sarmiento T Goddard LL Lordi V Gugov T Development of GaInNAsSb alloys: growth, band structure, optical properties and applications Phys Status Solidi B Basic Res 2007 9 2707 2729 10.1002/pssb.200675620

[B19] Dixit V Liu HF Xiang N Analysing the thermal-annealing-induced photoluminescence blueshifts for GaInNAs/GaAs quantum wells: a genetic algorithm based approach J. Phys Appl Phys 2008 9 115103 10.1088/0022-3727/41/11/115103

[B20] Liu HF Dixit V Xiang N Anneal-induced interdiffusion in 1.3-μmGaInNAs/GaAs quantum well structures grown by molecular-beam epitaxy J Appl Phys 2006 9 013503 10.1063/1.2150259

[B21] Sun Z Xu ZY Yang XD Sun BQ Ji Y Zhang SY Ni HQ Niu ZC Nonradiative recombination effect on photoluminescence decay dynamics in GaInNAs/GaAs quantum wells Appl Phys Lett 2006 9 011912 10.1063/1.2161071

[B22] Kudrawiec R Sęk G Misiewicz J Gollub D Forchel A Explanation of annealing-induced blueshift of the optical transitions in GaInAsN/GaAs quantum wells Appl Phys Lett 2003 9 2772 2774 10.1063/1.1615673

[B23] Lordi V Yuen HB Bank SR Wistey MA Harris JS Friedrich S Nearest-neighbor distributions in Ga_1-*x*_In_*x*_N_*y*_As_1-*y*_ and Ga_1-*x*_In_*x*_N_*y*_As_1-*y*-*z*_Sb_*z*_ thin films upon annealing Phys Rev B 2005 9 125309

[B24] Buyanova IA Chen WM Pozina G Bergman JP Monemar B Xin HP Tu CW Mechanism for low-temperature photoluminescence in GaNAs/GaAs structures grown by molecular-beam epitaxy Appl Phys Lett 1999 9 501 503 10.1063/1.124429

[B25] Kudrawiec R Sek G Misiewicz J Li LH Harmand JC Investigation of recombination processes involving defect-related states in (Ga, In)(As, Sb, N) compounds Eur Phys J Appl Phys 2004 9 313 316 10.1051/epjap:2004056

[B26] Kaschner A Lüttgert T Born H Hoffmann A Egorov AY Riechert H Recombination mechanisms in GaInNAs/GaAs multiple quantum wells Appl Phys Lett 2001 9 1391 1393 10.1063/1.1355014

[B27] Baranovskii SD Eichmann R Thomas P Temperature-dependent exciton luminescence in quantum wells by computer simulation Phys Rev B 1998 9 13081 13087 10.1103/PhysRevB.58.13081

[B28] Mair RA Lin JY Jiang HX Jones ED Allerman AA Kurtz SR Time-resolved photoluminescence studies of In_*x*_Ga_1-*x*_As_1-*y*_N_*y*_ Appl Phys Lett 2000 9 188 190 10.1063/1.125698

[B29] Zu LQ Lin JY Jiang HX Dynamics of exciton localization in a CdSe_0.5_S_0.5_ mixed crystal Phys Rev B 1990 9 7284 7287 10.1103/PhysRevB.42.7284 9994867

[B30] Ouadjaout D Marfaing Y Thermal activation of localized excitons in Zn_*x*_Hg_1-*x*_Te semiconductor alloys: photoluminescence line-shape analysis Phys Rev B 1992 9 7908 7910 10.1103/PhysRevB.46.7908 10002535

[B31] Cho Y-H Song JJ Keller S Minsky MS Hu E Mishra UK DenBaars SP Influence of Si doping on characteristics of InGaN/GaN multiple quantum wells Appl Phys Lett 1998 9 1128 1130 10.1063/1.122105

[B32] Cho Y-H Gainer GH Fischer AJ Song JJ Keller S Mishra UK DenBaars SP "S-shaped" temperature-dependent emission shift and carrier dynamics in InGaN/GaN multiple quantum wells Appl Phys Lett 1998 9 1370 1372 10.1063/1.122164

[B33] Lin YC Chung HL Chou WC Chen WK Chang WH Chen CY Chyi JI Carrier dynamics in isoelectronic ZnSe_1-*x*_O_*x*_ semiconductors Appl Phys Lett 2010 9 041909 10.1063/1.3473776

[B34] Gourdon C Lavallard P Exciton transfer between localized states in CdS_1–*x*_Se_*x*_ alloys Phys Status Solidi B 1989 9 641 652 10.1002/pssb.2221530222

[B35] Rubel O Baranovskii SD Hantke K Kunert B Rühle WW Thomas P Volz K Stolz W Model of temperature quenching of photoluminescence in disordered semiconductors and comparison to experiment Phys Rev B 2006 9 233201

[B36] Rubel O Galluppi M Baranovskii SD Volz K Geelhaar L Riechert H Thomas P Stolz W Quantitative description of disorder parameters in (GaIn)(NAs) quantum wells from the temperature-dependent photoluminescence spectroscopy J Appl Phys 2005 9 063518 063518–7 10.1063/1.2058192

[B37] Grüning H Kohary K Baranovskii SD Rubel O Klar PJ Ramakrishnan A Ebbinghaus G Thomas P Heimbrodt W Stolz W Rühle WW Hopping relaxation of excitons in GaInNAs/GaNAs quantum wells Phys Status Solidi C 2004 9 109 112 10.1002/pssc.200303604

[B38] Baranowski M Latkowska M Kudrawiec R Misiewicz J Model of hopping excitons in GaInNAs: simulations of sharp lines in micro-photoluminescence spectra and their dependence on the excitation power and temperature J Phys Condens Matter 2011 9 205804 10.1088/0953-8984/23/20/205804 21540495

[B39] Oueslati M Benoit C Zouaghi M Resonant Raman scattering on localized states due to disorder in GaAs_1-*x*_P_*x*_ alloys Phys Rev B 1988 9 3037 3041 10.1103/PhysRevB.37.3037 9944881

